# Anthropometric Factors on Safe Distances between Popliteal Vessels to the Femur for Cerclage Wiring of the Distal Femoral Fracture: A Magnetic Resonance Imaging Study

**DOI:** 10.3390/medicina56120655

**Published:** 2020-11-28

**Authors:** Hao-Wei Chang, Chia-Yu Lin, Hui-Yi Chen, Yi-Wen Chen, Hsien-Te Chen, I-Hao Lin, Chin-Jung Hsu, Tsung-Li Lin

**Affiliations:** 1 Department of Orthopedics, China Medical University Hospital, China Medical University, Taichung 40447, Taiwan; D22067@mail.cmuh.org.tw (H.-W.C.); D29585@mail.cmuh.org.tw (C.-Y.L.); D2326@mail.cmuh.org.tw (H.-T.C.); D24686@mail.cmuh.org.tw (I-H.L.); 2 Department of Radiology, China Medical University Hospital, China Medical University, Taichung 40447, Taiwan; D7396@mail.cmuh.org.tw; 3 Graduate Institute of Biomedical Sciences, China Medical University, Taichung 40447, Taiwan; T27330@mail.cmuh.org.tw; 4 3D Printing Medical Research Institute, Asia University, Taichung 41354, Taiwan; 5 Spine Center, China Medical University Hospital, China Medical University, Taichung 40447, Taiwan; 6 Department of Sport Medicine, College of Health Care, China Medical University, Taichung 40447, Taiwan; 7 School of Chinese Medicine, China Medical University, Taichung 40447, Taiwan

**Keywords:** popliteal vessel, vascular injury, magnetic resonance imaging, cerclage wiring, distal femoral fracture

## Abstract

*Background and Objectives*: The proximity of the popliteal vessels in the distal femur may increase the risk of iatrogenic vascular injury during cerclage wiring. In this study, the closest location and distance of the popliteal vessels to the femur was examined using magnetic resonance imaging (MRI). The associations between anthropometric factors and the distance that would guide the placement of wires safely during surgery were also identified. *Materials and Methods*: We reviewed adult knee magnetic resonance images and recorded: (1) the relation and the shortest horizontal distance (d-H) from the femoral cortex to the popliteal vessels in axial images and (2) the vertical distance (d-V) from the adductor tubercle to the axial level of the d-H values in coronal images. The effects of anthropometric factors (sex, age, body height, body weight, body mass index, thigh circumference, femoral length and femoral width) on these distances were analysed. *Results:* Analysis of 206 knee magnetic resonance images revealed that the closet locations of popliteal vessels were at the posteromedial aspect of the femur. The d-H and d-V were 7.38 ± 3.22 mm and 57.01 ± 11.14 mm, respectively, and were both shorter in women than in men (*p* < 0.001). Multivariate analysis identified thigh circumference and femoral length as the most influential factors for the d-H and d-V, respectively (*p* < 0.001). Linear regression demonstrated a strong positive linear correlation between the thigh circumference and the d-H and between the femoral length and the d-V (Pearson’s r = 0.891 and 0.806, respectively (*p* < 0.001)). *Conclusions:* The closet location and distance of the popliteal vessels to the femur provide useful information for wire placement during distal femoral fracture surgery while minimising the risk of vascular injury. Given that patients with a smaller thigh circumference and a shorter femoral length are more likely to have a smaller d-H and a shorter d-V, respectively, cautious measures should be taken in such cases.

## 1. Introduction

Cerclage wiring is one of the most common and effective fixation methods for distal femur fractures [1,2,3], especially in fractures with oblique-, spiral- or spiral wedge-type patterns on radiography with or without a prosthesis. These fractures are classified according to the following systems (Arbeitsgemeinschaft für Osteosynthesefragen (AO) Type 33-A1, AO Type 33-A2, AO Type 33-A3, Rorabeck type II and interprosthetic fracture) [4,5,6]. Unfortunately, irrespective of the open or percutaneous technique, there are as many as 7% of vascular complications associated with inadequate use of cerclage wires [1,7]. Indeed, the occlusion of major vessels by cerclage wires could result in severe issues, such as below-knee amputation [8].

The management of vascular injuries associated with distal femoral fracture surgery requires the awareness of the anatomy of the popliteal vessel and the distal femur and adequate wire placement. The superficial femoral artery (SFA) crosses from anterior-superior to posterior-inferior in the distal third of the femur and then exits from the adductor hiatus (AH) to become the popliteal artery (PA), which comes closely to the cortex [9].

The proximity of popliteal vessels to the distal femur may increase iatrogenic injury during the cerclage wiring of the distal femoral fracture. Few studies focused on the vascular structure or AH in the distal third of the femur. In two cadaveric studies, the area up to 8 cm proximal to the adductor tubercle (AT) was reported to be safe from vascular damage during surgery, and the localisation of the apex of the AH could be determined by a bony landmark [10]. However, the spatial resolution of the popliteal vessels in relation to the femur has not yet been examined, and the effects of the anthropometric factors on such measures have not been elucidated.

This study aimed to determine: (1) the closest location and distance of the popliteal vessels to the femur in adults on magnetic resonance imaging (MRI); and (2) the significance of associations between anthropometric factors (sex, age, body height, body weight, body mass index (BMI), thigh circumference, femoral length and femoral width) and the distance that would guide the placement of wires to minimise the risk of vascular injuries during distal femur fracture surgery.

## 2. Materials and Methods

### 2.1. Patients

After obtaining the institutional review board approval (CMUH109-REC3-106, date of approval: 10 August 2020), we conducted a retrospective review of consecutive knee MRI studies using our hospital’s database.

The inclusion criteria were patients between 20 and 80 years of age who underwent knee MRI in a 5-year interval (January 2015 to December 2019). Patients were excluded, if they had previous knee surgery, an implant in situ around the knee, soft tissue or bone tumours around the knee, infection around the knee or peripheral vessel disease. All studies had a written report submitted by a musculoskeletal radiologist (HYC) at our institution. All cases were on unilateral knees. The age of each patient at the time of the study was recorded. An electronic query and a manual review of the medical records were completed to obtain patient anthropometric factors, including sex, body height, body weight and thigh circumference, which was measured horizontally just distal to the gluteal fold [11]. BMI was calculated as the weight in kilograms divided by the square of the height in metres (kg/m^2^). The femoral length and width, defined as the distance from the tip of the greater trochanter (GT) to the AT and the widest portion of the distal femur, respectively [12], were reviewed on lower limb scanograms.

### 2.2. Knee MRI

Patients were scanned with a 3 Tesla (3T) Signa MRI scanner (General Electric Medical Systems, Milwaukee, WI, USA) in the supine position, with both lower extremities straight and knees extended. T1-weighted images in the axial, sagittal and coronal planes with a slice thickness of 2 mm were selected on each knee magnetic resonance image for analysis. The distances were measured using a digital calliper tool within INFINITT’s Picture Archiving and Communications System.

**Figure 1:**
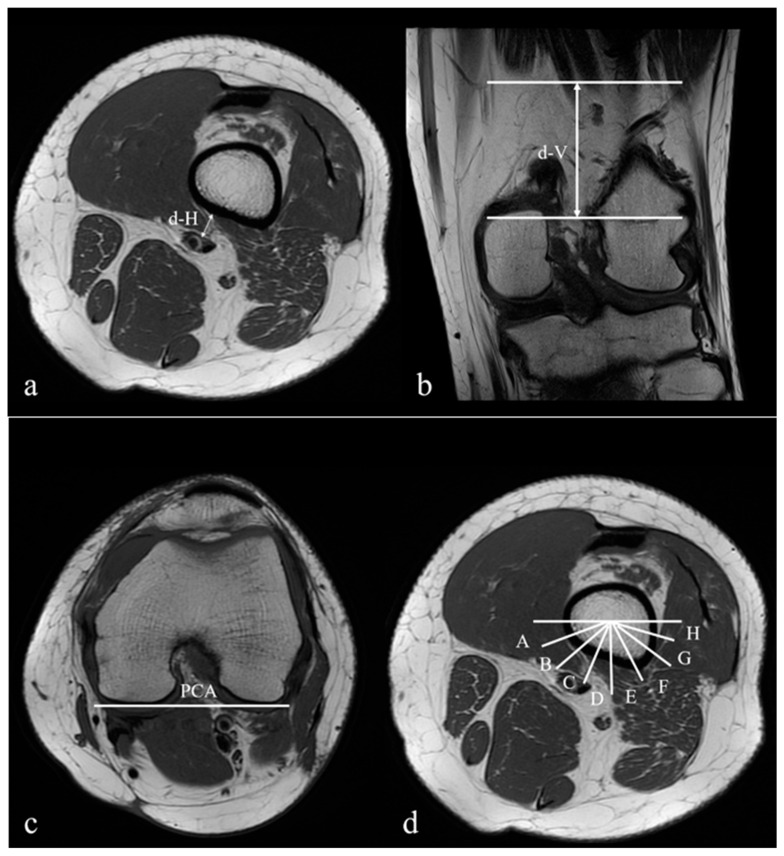
Magnetic resonance images demonstrating the views used for measuring the distances from the popliteal vessels to the femur: (**a**) in the axial views, the closest distance (d-H) between the popliteal vessels and the femoral cortex; (**b**) in the coronal views, the distance (d-V) between the adductor tubercle (AT) and the axial level of “d-H”; (**c**) in the axial views, the posterior condyles axis (PCA) in the femur used as a reference line (0°); (**d**) in the axial views, the posterior half of the femur divided into eight sections labelled from A to H and from posteromedial to posterolateral.

In the axial images, the shortest horizontal distance (d-H) from the femoral cortex to the popliteal vessels was measured after tracing the nearby cuts of the AH (Figure 1a). In the coronal images, the vertical distance (d-V) from the axial cut of the AT to the “d-H” axial level was measured (Figure 1b). The posterior condylar axis (PCA), a line connecting the most posterior border of the medial and lateral condyle in the axial view, was used as a reference to set the sagittal plane of the femur (Figure 1c). The posterior half of the femur was defined by a line paralleling the PCA and crossing the centre of the femoral canal, as described by Kim et al. [13]. At each “d-H” axial level, the posterior half of the femur was divided into eight sections labelled “A” to “H” from posteromedial to posterolateral, and the position of the popliteal vessels was noted (Figure 1d).

**Figure 2:**
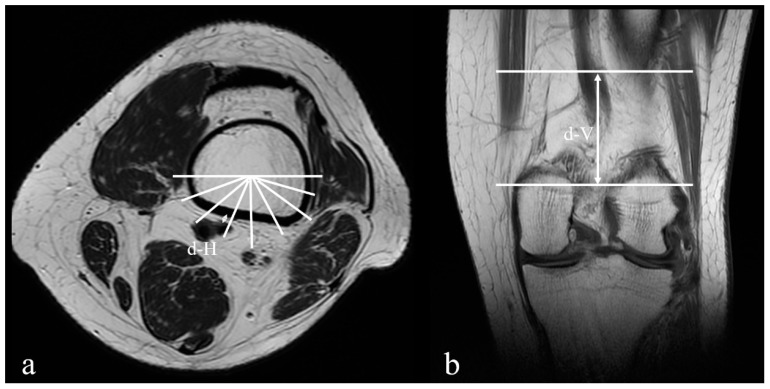
The example of the measurements in magnetic resonance images of a 28-year-old male: (**a**) the closest distance (d-H) between the popliteal vessels and the femoral cortex is 1.22 mm, and the position of the popliteal vessels adjacent to the femoral cortex is section C; (**b**) the distance (d-V) between the AT and the axial level of “d-H” is 66.98 mm.

**Figure 3:**
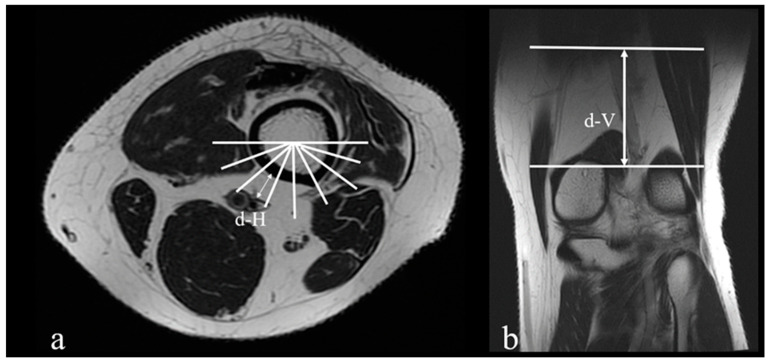
The example of the measurements in magnetic resonance images of a 56-year-old female: (**a**) the closest distance (d-H) between the popliteal vessels to the femoral cortex is 14.65 mm, and the position of the popliteal vessels adjacent to femoral cortex is section C; (**b**) the distance (d-V) between the AT and the axial level of “d-H” is 41.08 mm.

One musculoskeletal radiologist (HYC) and two orthopaedic surgeons (HWC and TLL) recorded all measurements independently, and the mean between three physicians was used for data analysis. Figure 2 and Figure 3 illustrate the examples of the measurements in MRI.

### 2.3. Statistical Analysis

Power analyses [14] with G*Power 3.1 (Franz Faul, Universitat Kiel, Germany) revealed that a minimum sample size of 109 was necessary to detect medium effect sizes (f^2^ ≥ 0.15) with a power of 0.80 and α of 0.05, and eight predictors. Statistical analyses were performed using SPSS for Windows, version 21.0 (SPSS Inc., Chicago, IL, USA). The reliability of each measurement was examined by the intra-class correlation coefficient (ICC). Continuous data are presented in the form of mean ± standard deviation. Groups were compared using a *t*-test for independent samples. The effects of sex, age, body height, body weight, BMI, thigh circumference, femoral length and femoral width on each measurement were evaluated using multivariate linear regression analysis. The coefficient of determination, R^2^, was used to check the goodness of fit of the statistical models, and the original uncertainty in the data was explained by the multivariate analysis. R^2^ varied between 0 and 1, with 0 indicating no benefit and 1 indicating benefit gained by applying multivariate analysis. The correlation between the most influential anatomical factor and the distance measurements was analysed using Pearson’s correlation coefficient, and significant differences were examined with Games-Howell post-hoc analysis. Statistical significance was set at *p* < 0.05.

## 3. Results

A total 206 consecutive MRI scans of the knee were analysed with the following diagnosis: ligament and meniscus lesions (*n* = 117), osteoarthritis (*n* = 47), spontaneous osteonecrosis of the knee (N = 23) and osteochondritis dissecans (*n* = 19).

The study group included 110 men and 96 women with a mean age of 47.55 years (range 20–80 years), a mean height of 165.53 cm (range: 138–188 cm), a mean body weight of 69.62 kg (range: 39–140 kg), a mean BMI of 25.24 (range: 16.92–40.17), a mean thigh circumference of 479.33 mm (range: 360–649 cm), a mean femoral length of 413.85 mm (range: 330.16–479.84 mm) and a mean femoral width of 84.17 mm (range: 64.13–100.41 mm).

The knee MRI included 113 right and 93 left sides. There was no significant difference between the right and left sides according to sex (*p >* 0.05). The position of the popliteal vessels adjacent to the femoral cortex was section C (109/206, 52.9%), followed by sections B, D and A (27.2%, 19.4% and 0.5%, respectively). The ICCs of the d-H and the d-V were 0.915 (range: 0.883–0.947) and 0.923 (range: 0.897–0.961), respectively. The d-H was 7.38 ± 3.22 mm. The d-V was 57.01 ± 11.14 mm. There was no significant difference in the distances between the groups of different pathologic diagnoses of the knees (*p =* 0.721).

There was a significant difference between men and women in their d-H and d-V values as well as body height, body weight, BMI, thigh circumference, femoral length and femoral width (*p <* 0.001, *p* < 0.001, *p* = 0.008, *p* = 0.002, *p* < 0.001 and *p* < 0.001, respectively; Table 1), Because the sex difference might account for changes in d-H and d-V, multivariate analysis was performed.

The results are shown in Table 2. The total effects (R^2^) of these anthropometric factors on the d-H and the d-V were 0.788 and 0.667, respectively. The d-H correlated with thigh circumference (*p <* 0.001) but not with sex, body height, body weight, BMI, femoral length or femoral width. The d-V correlated with femoral length (*p <* 0.001) but not with sex, body height, body weight, BMI, thigh circumference or femoral width.

**Figure 4:**
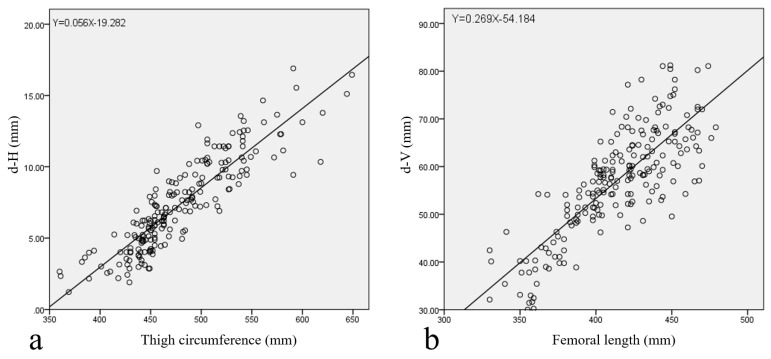
(**a**) The simple linear regression model describing the relationship between thigh circumference and d-H; (**b**) the simple linear regression model describing the relationship between femur length and d-V.

The linear regression equations predicting d-H (Figure 4a) and d-V (Figure 4b) were as follows:

d-H (mm) = 0.056 × thigh circumference − 19.282,(1)

d-V (mm) = 0.269 × femoral length − 54.184.(2)

Equations (1) and (2) predicted that patients with a smaller thigh circumference (especially smaller than 399 mm) had a smaller d-H (Table 3) and those with a shorter femoral length (especially smaller than 369 mm) had a shorter d-V (Table 4).

There was no significant difference between the measurements and age or BMI (Table 5 and Table 6).

## 4. Discussion

The proximity of the vascular structures traversing the AH in the distal femur may increase the risk of iatrogenic popliteal vascular injury during cerclage wiring. In the current study, the reference values for safe distances from the injury and the closest location of the popliteal vessels to the femur were established using MRI in adult knees. The closest locations of the popliteal vessels were at the posteromedial aspect of the femur. The d-H and the d-V were 7.38 ± 3.22 mm and 57.01 ± 11.14 mm, respectively. We also assessed the effect of anthropometric factors on these distances and found thigh circumference and femoral length to be the most important indicators for the d-H and the d-V, respectively.

Distal femur fractures account for approximately 6% of all femoral fractures [15,16,17], while vascular injuries account for approximately 2% [18]. Injuries to the SFA, deep femoral artery or PA have been described as a result of broken sharp fragments or iatrogenic injuries such as external fixation pins, plunging drill bits, medial plating or cerclage wiring. These types of damage could give rise to immediate bleeding, late presented pseudoaneurysm, limb ischaemia or below-knee amputation [1,8,19,20,21].

Apivatthakakul et al. evaluated the computed tomography angiography (CTA) images of 80 patients, which divided the whole femur into eight equal segments (seven levels) from the tip of the GT to the lateral tibiofemoral joint line in the coronal plane and eight equal directions from the anterior to the posterior of the medial femur in the axial plane. They have found that when the SFA is at levels 6 and 7, it is located between sectors F and H (posteromedial and posterior to the femur) and at a distance of approximately 13.63 ± 3.59 mm and 10.08 ± 3.09 mm, respectively [22]. Their result was similar to that of the current study, which revealed the closest point of the popliteal vessels was posteromedial and posterior to the femur. During cerclage wiring, either from the anterolateral or posterolateral direction, surgeons should be cautious of the posteromedial and posterior aspects of the femur. The present study demonstrated the precariousness of the popliteal vessels and that any distance shorter than the closest one shown here between the vessels and the femur cortex could prove more detrimental than previously thought.

To our best knowledge, this was the first study of d-H, which demonstrated a smaller d-H in patients results in a small thigh circumference. The explanation for this association is still uncertain. While the influence of obesity on the anatomical relationship between the PA and the tibial nerve in the popliteal fossa was reported, no direct evidence for the relationship between the popliteal vessels and the femur cortex was provided [23]. Chuckpaiwong et al. have found that the infrapatellar fat pad volume is correlated with age in the osteoarthritic group by 3T MRI, but not in the control subjects [24]. Song et al. have demonstrated that the popliteal fossa fat brook has no association with age [25]. Even though we hypothesised that d-H was related to the thickness of the fatty tissue around the popliteal fossa, no correlation between the measurement and age or BMI was noted. Therefore, body fat percentage and regional distribution should be included for evaluation in future studies.

There is a transition zone in the hiatal area from the adductor canal to the popliteal fossa. In comparison with the more flexible fatty tissue of the popliteal fossa, the AH region is more rigid and fixes the junction of the SFA and the PA close to the femur cortex [10]. Kwon et al. reported the AH level to be over 59.8 mm proximal to the superior border of the patella [26]. Cadaveric studies with 24 and 28 thighs described the level of AH to be above 10 cm (range: 8.0–13.5 cm) and 7.4 cm (range: 5.6–9.2 cm) from the AT, respectively [27,28]. Narulla et al. assessed 41 limbs using CTA to describe the relationship between the SFA and the whole femoral shaft and warned of the “danger zone” from 239.6 mm to 172.5 mm proximal to the AT [12]. In the current study, the d-V (57.01 ± 11.14 mm) was shorter than the distance between the AT and the AH that was described in the literature [27,28]. This suggests that the closest level of the vascular bundle occurs slightly distal to the AH at the point where the PA crosses posteriorly to the distal femur.

All studies of femoral vessels around the distal femur that have been found in the literature are summarised in Table 7. The trend is for a higher position of the danger zone of the femoral vessels proximal to the AT and larger distances from the FA to the femoral cortex in patients from the USA and Australia than in patients from Korea, Thailand, Taiwan and other Asian countries. These differences might be explained by racial variations in the anatomy of femoral vessels and femoral bones or by different femoral lengths and thigh circumferences between the races. However, because the studies did not record the race of the patients, and because of the variety of study designs, the racial influence, if any, remains unclear.

Narulla et al. proposed the doubled width of the femoral condyles as an estimated safe distance proximal to the AT for intervention [12]. They also mentioned the danger zone in which the SFA crossed inferiorly by halving the distance between the GT and the AT. Both predictors could be measured on a true anteroposterior (AP) radiograph preoperatively or intraoperatively. In the current study, the surgeon could estimate d-H and d-V preoperatively based on the thigh circumference and the femoral length, respectively, without CTA or MRI. The thigh circumference could easily be computed directly below the gluteal fold [11]. The femoral length could be calculated from the length between the GT and the AT on the AP view of a whole femur radiograph. The thigh circumference and the femoral length could be used clinically by measuring the normal contralateral instead of the fractured limb.

The important clinical implications of the current study were that it found the closest location and distance of the popliteal vessels to the femur, to provide useful information for wire placement during distal femoral fracture surgery while minimising the risk of vascular injury. Surgeons should strive to perform subperiosteal dissection and pass the wire passer tips as close to the bony cortex as possible during wiring to avoid vascular injury based on the patient’s thigh circumference. The vertical positions of the cerclage wire should be checked intraoperatively based on the AT and the femur length to estimate the low-risk position from the popliteal vessels. Given that patients with smaller thigh circumferences and shorter femoral lengths are more likely to have a smaller d-H and a shorter d-V, respectively, caution should be taken in such cases.

### 4.1. Limitations

This study has several limitations. First, all magnetic resonance images were performed to diagnose the pathology. None of them were performed on a strictly defined normal population. However, we excluded patients whose vessels were potentially affected by the pathology to minimise the effects on the data. Second, our distances were measured on magnetic resonance images, not intraoperatively. Third, given that the magnetic resonance images were taken with the patient in a supine position, which was similar to most of the clinical conditions that used the supine lateral approach, while some surgeries were performed with the patient in a lateral decubitus position in the distal femur fracture and certain anatomical relationships may differ. Moreover, the position of vessels could change with bending, torsion and compression in the flexed knee [30]. Further study with different groups of lower limb positions will be needed. Fourth, instead of measuring the distances on 2D MRI scans, it would be more reliable to make 3D reconstructions of the soft tissue of interest, i.e., the vessels, using software such as Mimics^®^ (Materialise, Leuven, Belgium) and relate those distances to the 3D bony landmarks. Last, given that all subjects presented here were of the unrelated Han Chinese ethnicity, it would be interesting to conduct this work in populations of different races.

### 4.2. Strengths

Several methods of evaluating the location of the femoral artery have been utilised including cadaveric dissection [10,28,31], ultrasonography, angiography [18,32], CTA [9,12,13,21,22,33] and MRI [34,35,36]. We believe MRI provides more valuable information about relationships between soft tissues, including the neurovascular bundle, muscles and fatty tissues. In addition, the innovation of the noncontrast-enhanced MRI technique improved the resolution of soft tissue anatomy without the risk of contrast-induced complication as seen in angiography or CTA [34,37]. Moreover, the sample size in our study was large, and the analysed magnetic resonance images were used to assess structurally pathologic knees, which was reflective of the anatomical reality in patients who underwent surgery. All distances were independently measured by three physicians. Therefore, the results provided could be viewed as reliable reference data for future work. Moreover, the aim of the study was to determine: (1) the closest location and distance of the popliteal vessels to the femur on MRI and (2) the association between anthropometric factors and the distance that would guide the placement of wires to minimise the risk of vascular injuries during distal femur fracture surgery. MRI was not considered necessary preoperatively, and the ultrasonography of the limb prior to surgery was encouraged because it provided the same information more quickly than MRI, especially in vulnerable patients with smaller thigh circumferences.

## 5. Conclusions

By measuring a large number of adult knee magnetic resonance images, this study found the closest location and distance of the popliteal vessels to the femur to provide useful information for wire placement during distal femoral fracture surgery while minimising the risk of vascular injury. Surgeons should strive to perform subperiosteal dissection and pass the wire passer tips as close to the bony cortex as possible during wiring to avoid vascular injury based on the patient’s thigh circumference. The vertical positions of the cerclage wire should be checked intraoperatively based on the AT and the femur length to estimate the low-risk position from the popliteal vessels. Given that patients with a smaller thigh circumference and a shorter femoral length are more likely to have a smaller d-H and a shorter d-V, respectively, caution should be taken in such cases.

## Figures and Tables

**Table 1:** Sex differences in anthropometric factors, d-H and d-V.

	Men (*n* = 110)	Women (*n* = 96)	*p* Value
Age (years)	44.98 ± 15.72	44.84 ± 17.46	0.952
BH (cm)	172.25 ± 6.90	157.74 ± 8.44	<0.001
BW (kg)	77.47 ± 16.50	60.58 ± 11.71	<0.001
BMI (kg/m^2^)	25.99 ± 4.59	24.33 ± 4.24	0.008
TC (mm)	489.58 ± 55.18	467.57 ± 44.36	0.002
FL (mm)	432.16 ± 25.97	392.86 ± 28.31	<0.001
FW (mm)	88.61 ± 5.81	78.96 ± 6.25	<0.001
d-H (mm)	7.92 ± 3.42	6.76 ± 2.86	0.010
d-V (mm)	61.79 ± 9.39	51.54 ± 10.49	<0.001

Data are presented as mean ± standard deviation. BH, body height; BW, body weight; BMI, body mass index; TC, thigh circumference; FL, femoral length; FW, femoral width; d-H, the closest horizontal distance between popliteal vessels and the femoral cortex; d-V, the vertical distance between AT and the axial level of d-H.

**Table 2:** Multivariate analyses of the distances of the popliteal vessels to the femur.

Distance	B Estimate	SE	*p* Value	R^2^
d-H				0.788
Intercept	−22.378	8.662		
Sex	−0.015	0.295	0.960	
Age (years)	0.000	0.007	0.986	
BH (cm)	0.024	0.054	0.655	
BW (kg)	−0.025	0.058	0.673	
BMI (kg/m^2^)	0.061	0.163	0.711	
TC (mm)	0.056	0.003	<0.001	
FL (mm)	−0.003	0.006	0.676	
FW (mm)	0.027	0.018	0.126	
d-V				0.667
Intercept	−90.768	37.717		
Sex	−2.513	1.286	0.052	
Age (years)	0.039	0.029	0.181	
BH (cm)	0.395	0.234	0.094	
BW (kg)	−0.094	0.253	0.712	
BMI (kg/m^2^)	0.557	0.712	0.435	
TC (mm)	−0.026	0.013	0.068	
FL (mm)	0.210	0.028	<0.001	
FW (mm)	0.147	0.077	0.057	

SE, standard error.

**Table 3:** The closest horizontal distances between the popliteal vessels and femoral cortex based on thigh circumference.

	Thigh Circumference (mm)					*p* Value
	≤399 (*n* = 8)	400–449 (*n* = 55)	450–499 (*n* = 79)	500–549 (*n* = 48)	≥550 (*n* = 16)	
d-H (mm)	2.93 ± 1.01	4.37 ± 1.31	6.95 ± 1.66	10.35 ± 1.55	13.10 ± 2.24	<0.001

Data are presented as mean ± standard deviation.

**Table 4:** The vertical distance between the AT and the d-H level based on femoral length.

	Femoral Length (mm)					*p* Value
	≤369 (*n* = 26)	370–399 (*n* = 37)	400–429 (*n* = 70)	430–459 (*n* = 55)	≥460 (*n* = 18)	
d-V (mm)	38.55 ± 6.40	49.76 ± 5.76	58.97 ± 57.51	64.65 ± 8.15	67.62 ± 6.63	<0.001

Data are presented as mean ± standard deviation.

**Table 5:** The closest horizontal distance between the popliteal vessels and the femoral cortex based on age stratified analysis.

	Age Stratification (Years)				*p* Value
	20–34 (*n* = 66)	35–49 (*n* = 56)	50–64 (*n* = 56)	65–80 (*n* = 28)	
d-H (mm)	7.70 ± 3.11	7.70 ± 3.57	6.97 ± 3.03	6.74 ± 3.01	0.360

Data are presented as mean ± standard deviation.

**Table 6:** The closest horizontal distance between the popliteal vessels and the femoral cortex based on BMI stratified analysis.

	BMI Stratification				*p* Value
	<18.5 (*n* = 9)	18.5–24.9 (*n* = 94)	25.0–29.9 (*n* = 76)	≥30 (*n* = 27)	
d-H (mm)	6.24 ± 3.26	7.14 ± 3.51	7.06 ± 3.68	7.37 ± 3.12	0.125

Data are presented as mean ± standard deviation.

**Table 7:** Summary of published studies of femoral vessels around the distal femur.

Study	Method	Numbers	Sex (M/F)	Age (Range)	Country	Finding
Olson et al. [27]	Cadaver	24	13/11	80 (60–98)	USA	The danger zone of the femoral vessels was 80–135 mm (mean: 100 mm) proximal to the AT. The distances from the femoral vessels to the femoral cortex were NA.
Maslow et al. [9]	CTA	30	8/7	50 (25–80)	USA	The danger zone of the SFA was 142.6 ± 40.6 mm proximal to the AT. At that level, the distances from the SFA to the medial femoral cortex were 30.7 ± 8.7 mm.
Narulla et al. [12]	CTA	41	16/6	60.5 (51–89)	Australia	The danger zone of the SFA was 172.5 ± 40.9 mm proximal to the AT. At that level, the distances from the SFA to the femoral cortex were 23.0 mm to 26.7 mm.
Kim et al. [13]	CTA	30	18/12	52.4 (24–73)	Korea	The femur was divided into six levels from the LT to the AT (1–6). The distances from the FA to the femoral cortex were 7.5–18.3 mm (mean: 12.2 mm) at level 6.
Jiamton et al. [29]	CTA of cadaver	20	NA	NA	Thailand	The femur was divided from the GT to the knee joint line into seven levels (1–7). The distances from the SFA to the medial femoral cortex were 8.3–32.8 mm (mean: 18.2 mm) at level 6.
Apivatthakakul et al. [22]	CTA	80	27/13	51.6 (21–70)	Thailand	The femur was divided from the GT to the knee joint line into seven levels (1–7). The distances from the SFA to the femoral cortex were 13.63 ± 3.59 mm and 10.08 ± 3.09 mm at levels 6 and 7, respectively.
Chang et al. (current study)	MRI	206	110/96	47.55 (20–80)	Taiwan	The danger zone of the popliteal vessels was 57.01 ± 11.14 mm proximal to the AT. At the level, the distance from the popliteal vessels to the medial femoral cortex was 7.38 ± 3.22 mm.

CTA, computed tomography angiogram; GT: greater trochanter; LT, lesser trochanter; FA: femoral artery; SFA: superficial femoral artery; MRI, magnetic resonance imaging; NA, not available.
